# The efficiency of patient-specific instrumentation and technological assistance in cementless total hip arthroplasty via the direct anterior approach

**DOI:** 10.1186/s12893-025-02950-5

**Published:** 2025-05-15

**Authors:** Laurentiu Cosmin Focsa, Giacomo Galanzino, Philippe Gerard, Vincent Le Strat, Luc Lhotellier, Thomas Aubert

**Affiliations:** Groupe Hospitalier Diaconesses Croix Saint-Simon, Paris, France

**Keywords:** Patient-specific instrumentation, Total hip arthroplasty, Personalized surgery, 3D plan, Anterior approach of the hip, Surgical time

## Abstract

**Background:**

Patient-specific instrumentation for total hip arthroplasty (PSI-THA) is an emerging technology that improves the accuracy of femoral neck osteotomy and implant positioning. Unlike conventional 2D radiograph-based planning, PSI-THA leverages 3D CT reconstructions for personalized, technology-assisted positioning. This study sought to assess the intraoperative efficiency of PSI-THA in terms of surgery duration and blood loss by comparing PSI incorporating image-based guides and 3D planning with conventional surgery and 2D planning for cementless THA performed via the direct anterior approach (DAA).

**Methods:**

Two consecutive cohorts of 100 patients each were retrospectively analysed. All patients underwent cementless THA with a straight quadrangular stem and a ceramic-on-ceramic head and liners. Two-dimensional templating was performed for the first cohort, whereas a 3D template with CT-based PSI for femoral neck osteotomy and acetabular cup positioning was performed for the second cohort. A laser guidance system was employed to increase implant placement accuracy. Operating time and intraoperative blood loss were compared between the groups.

**Results:**

The demographic characteristics of the two groups were comparable. The average operating time was 45.7 min (SD: 16.11) in the conventional group and 31.9 min (SD: 9.92) in the PSI group (*p* < 0.001). Blood loss was also significantly lower in the PSI group (319 ml) than in the conventional group (407 ml; *p* < 0.017).

**Conclusions:**

Compared with conventional planning, PSI with 3D planning and technological assistance significantly reduced the operating time by an average of over 10 min as well as the amount of blood loss. The improved planning and execution accuracy of PSI minimizes the need for intraoperative adjustments, improves confidence in implant positioning, and reduces the need for compromises and the identification of multiple landmarks, underscoring the value of this planning technology in DAA THA.

**Clinical trial number:**

Not applicable.

## Background

Implant placement in total hip arthroplasty (THA) is crucial for achieving optimal functional outcomes and minimizing patient complications. Incorrect positioning can lead to complications such as impingement, dislocation, restricted range of motion, accelerated wear, and ultimately implant failure, often leading to the need for revision surgery [1–3].

Although two-dimensional (2D) templating is the current gold standard, transitioning to three-dimensional (3D) preoperative planning for cementless THA could lead to important benefits, including greater accuracy in selecting the patient’s implant, improved alignment and offset, and a reduction in postoperative complications [4–8]. The use of 3D planning allows better anticipation of the prosthesis size, thus avoiding undersizing, which could lead to subsidence or stem varus [9], affecting the prosthetic offset [10]. Conversely, 3D planning also helps prevent stem oversizing, which increases the risk of femoral fracture. Furthermore, the placement of the femoral stem influences the leg length and the osteotomy is often planned according to the distance from the lesser trochanter as determined by preoperative planning. Compared with freehand techniques, the use of custom-made guides improves the accuracy of femoral osteotomy, resulting in a precision of less than 3 mm [11, 12].

The positioning of the acetabular cup is one of the most demanding aspects of THA, exhibiting greater variability than that of other parts of the implant [13]. The Lewinnek safe zone (LSZ), defined as an inclination/anteversion of 40°/15° (± 10°) measured on postoperative supine radiographs [14], has long served as a benchmark for optimal acetabular cup positioning. However, evidence suggests that relying solely on these parameters may be insufficient, as 58% of patients with a history of dislocation have their acetabular components placed in the LSZ [15, 16]. Recent research has highlighted the importance of patient-specific safe zones that consider individual anatomical and functional characteristics [17]. The hip-spine classification system provides a framework for categorizing patients undergoing THA on the basis of spinopelvic pathologies. This approach enables the surgeon to stratify the patient by dislocation risk and refine the surgical plan to improve outcomes [18]. However, current techniques reliably achieve broad, ± 15° targets, and when traditional freehand techniques are used, success rates for achieving the target ranges in both inclination and anteversion have been reported to be as low as 20% and generally approximately 50% [19, 20]. Compared with conventional methods, techniques such as augmented reality (AR), computer-assisted navigation systems (CASs), patient-specific instrumentation (PSI), portable accelerometer-based navigation (PN) and laser guidance have been shown to increase the orientation accuracy of the cup [21]. However, these techniques, particularly robotic assisted systems (RASs) and CASs, are associated with increased surgical times and costs [22–24]. PSI based on 3D printing technology and image-based patient-specific guides is an innovative approach for increasing femoral neck osteotomy precision and improve implant positioning accuracy in THA while avoiding the time demands of navigated or robotic surgical techniques. Furthermore, compared with conventional methods, the relatively novel PSI-based surgical technique achieves greater precision in acetabular and cup prosthesis positioning, optimizes the surgical procedure, reduces complications, and promotes faster hip function recovery postsurgery in adults with Crowe III and IV developmental dysplasia of hip (DDH)-THA [25].

Reducing operative time and intraoperative blood loss are important factors in total hip arthroplasty (THA). Shorter surgical duration has been associated with lower rates of perioperative complications, including reduced risks of infection, thromboembolic events, and anesthesia-related morbidity [26]. Furthermore, minimizing blood loss decreases the need for postoperative blood transfusions, which may be associated with immunologic reactions and prolonged hospital stays [27].

The aim of this study was to evaluate the intraoperative efficiency of PSI and 3D planning, specifically focusing on operating time and blood loss during THA, and compare it to conventional surgery based on 2D planning for cementless THA performed via the direct anterior approach (DAA).

## Methods

### Population

Two consecutive cohorts of 100 patients each were retrospectively analysed following THA at the same hospital between November 2021 and December 2022. The use of 3D planning depended on the limited daily availability of the dedicated instrumentation required for the OPS system, without any patient selection for either method.

**Fig. 1:**
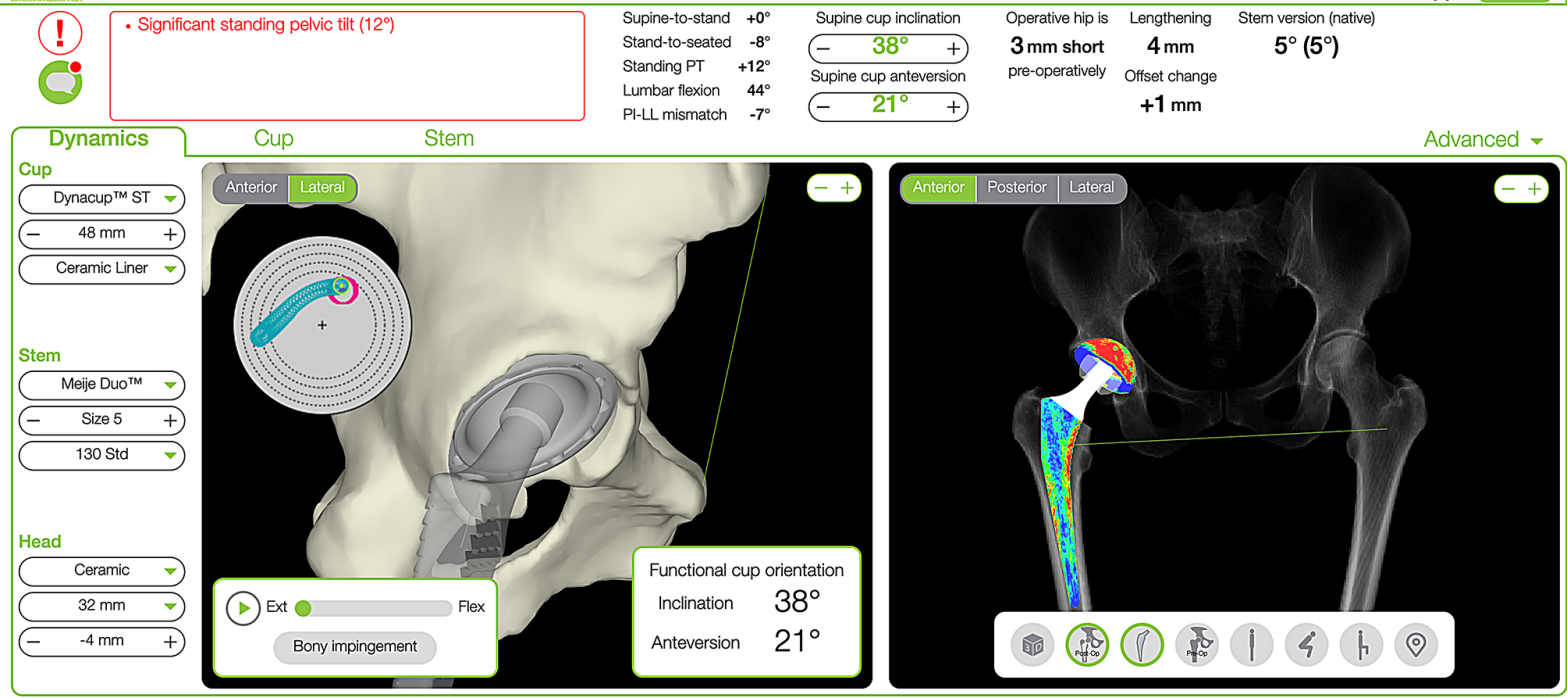
Preoperative implant positioning planning via 3D CT reconstruction. Distribution of the load sharing at the bone–implant interface (right)

All patients underwent THA performed by three senior surgeons with a cementless straight quadrangular stem with a ceramic-on-ceramic head and liners (Meije Dynacup; Corin, Cirencester, UK). Planning in the conventional group was performed via 2D X-rays in MediCAD software (Hectec GmbH, Germany), whereas that in the PSI group was performed via 3D CT imaging in OPSInsight software (Corin, UK) (Fig. 1). The data of the patients in these cohorts were previously analysed in a publication that compared the accuracy of 2D digital and 3D CT-constructed plans for selecting implant size and prosthetic offset for the same cementless prosthesis via an anterior approach. In contrast, the present publication focuses on technical issues and the effectiveness of the surgical procedure [28].

Each surgeon performed the operations were performed via the DAA with patients in the supine position on a traction table. Fluoroscopy was not performed.

Radiological assessments were independently performed twice by two different examiners. The study received approval from the local ethics committee, and all patients provided informed consent.

### Surgical planning and surgical technique

**Fig. 2:**
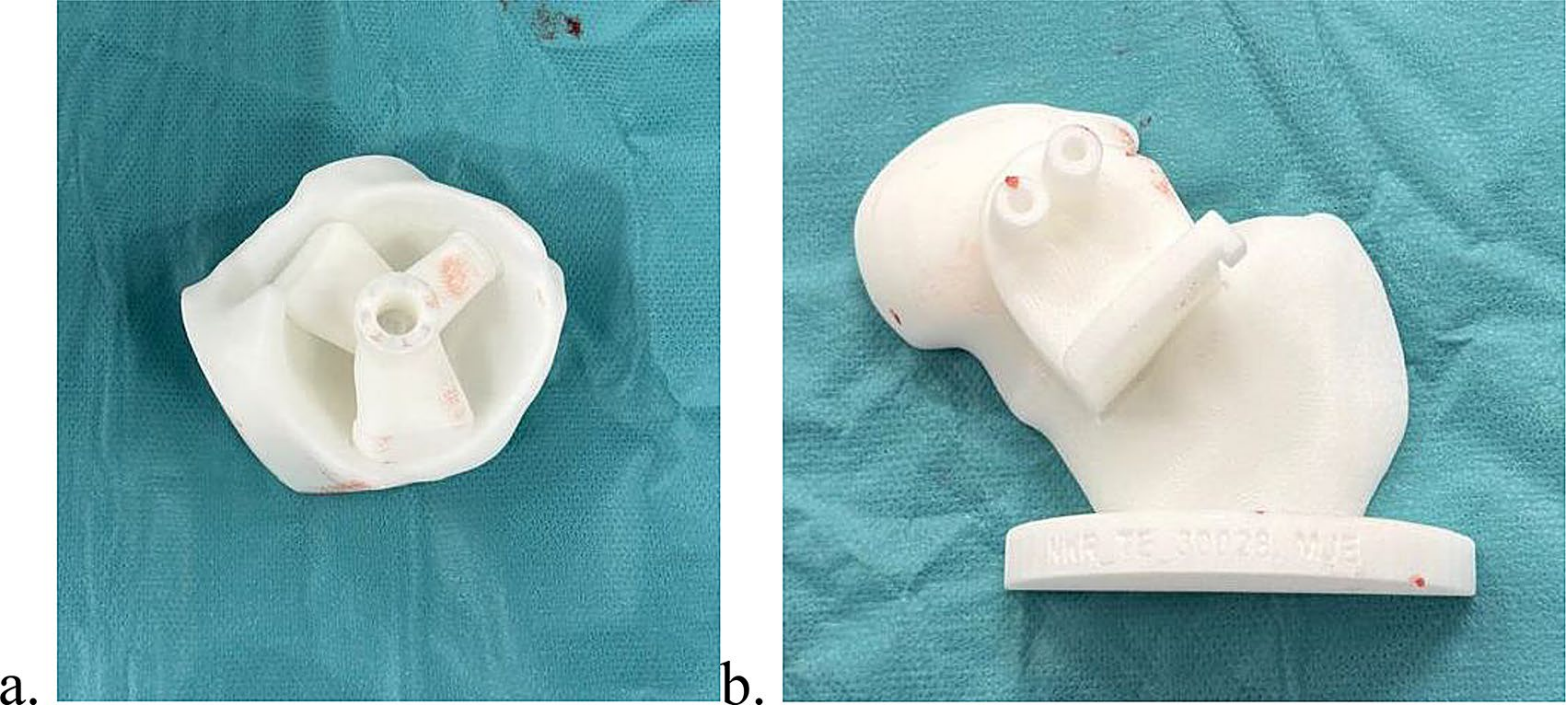
3D patient-specific guides. **a**. Guide for acetabular implant positioning and b. guide for performing femoral neck osteotomy

Patients included in the PSI group underwent low-dose CT scans (2.8–4.0 mSv) as part of the OPS Dynamic Hip Analysis Protocol (Optimized Ortho, Corin, Sydney) [29]. The range of the scan encompassed the entire bony pelvis, extending from the top of the iliac crest to 20 cm distal to the centre of the femoral head, with a 1.25 mm slice thickness. Using ScanIP v5.1 (Simpleware, Exeter, United Kingdom), 3D reconstructions of the pelvis were created, and the anterior superior iliac spines and pubic prominences were identified to define the anterior pelvic plane (APP) and determine the optimal acetabular implant position. The position of the acetabular cup, in terms of inclination and anteversion, was determined based on femoral version and the analysis of spinopelvic mobility from standing to sitting position, in order to achieve the optimal orientation to minimize the risk of prosthetic impingement and edge loading. Patient-specific guides were designed and 3D printed for precise acetabular positioning on the basis of the 3D planning (Fig. 2a). Additionally, patient-specific guides were used for the femoral neck osteotomy (Fig. 2b).

Four additional radiographs were obtained for the patients in the PSI group: one in a standing AP pelvic view and three in lateral views of the entire lumbar spine and pelvis, taken in three different positions—flexed seated, standing and standing with 90° flexion of the contralateral hip—as part of the OPS protocol [30].

**Fig. 3:**
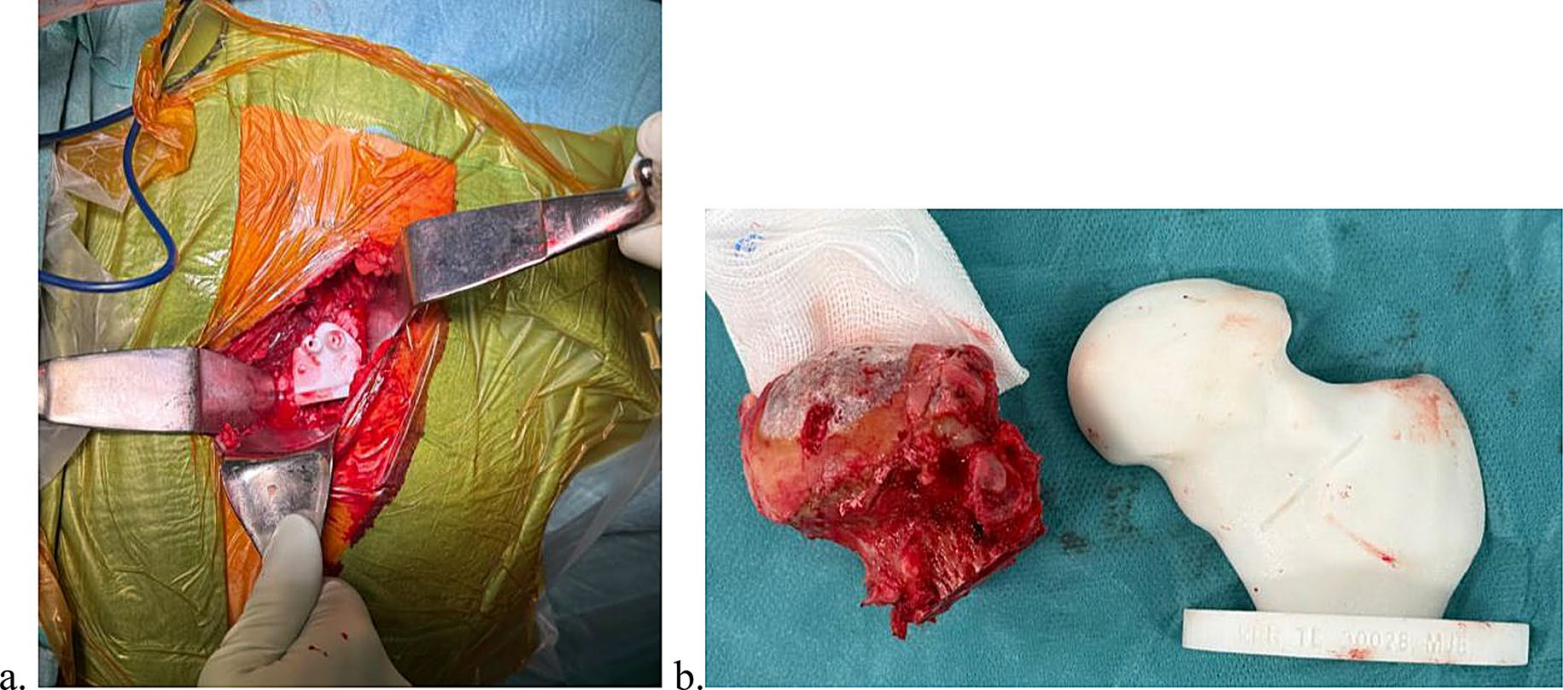
Patient-specific femoral neck guide **a**. Placed intraoperatively. **b**. Height of the femoral neck osteotomy

The goal was to reestablish the femoral offset and the height of the femoral head centre from the top of the greater trochanter. The custom femoral neck guide was placed after the articular capsule was excised to perform the osteotomy (Fig. 3).

**Fig. 4:**
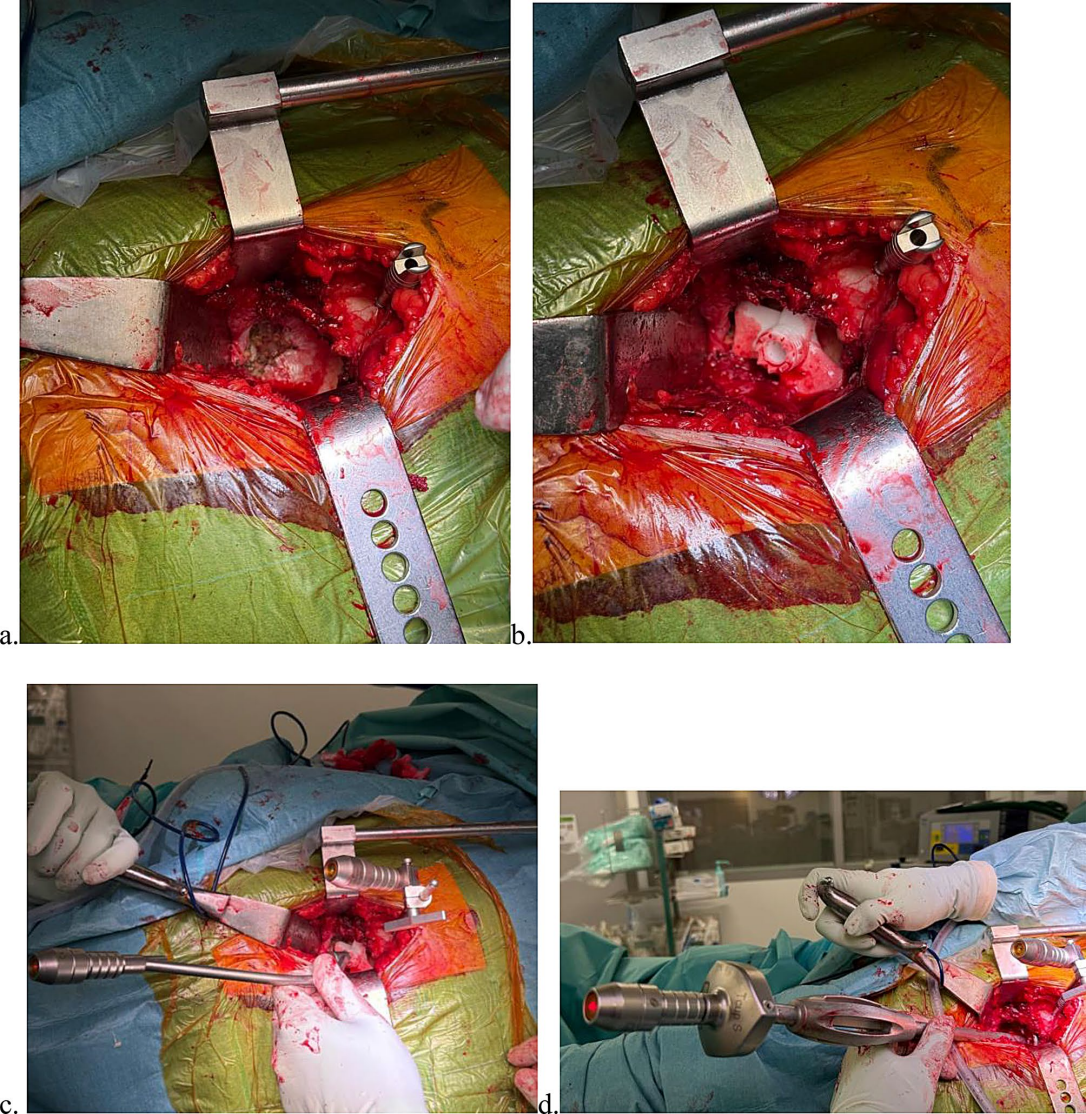
Positioning of the laser guide **a**. Proper preparation of the acetabular fossa. **b**. Patient-specific acetabular guide positioned according to the preoperative plan, with installation of the pelvic landmark. **c**. Pelvic landmark with an attached laser beam emitter whose beam converges with the guide laser at a single point on the operating room wall. **d**. Positioning of the acetabular component according to the preoperative 3D plan. The introducer laser beam converges with the pelvic landmark laser at a single point on the operating room wall

After exposing the implant area, a custom acetabular implant was inserted into the cavity until stability was achieved. The patient-specific 3D-printed acetabular positioning guide, created from the CT scan, allows for stable placement on the bony landmarks of the acetabular cavity. A laser guide mounted on the 3D guide enables the projection of a light dot onto the operating room wall, defining the desired orientation for the acetabular component. A second laser, mounted on a screw fixed to the pelvis, was positioned so that the projections from the custom guide and the pelvic reference laser converged at a single point on the operating room wall. This stationary pelvic laser was then secured and used to monitor any intraoperative pelvic position shifts. The guide was then removed from the acetabulum (Fig. 4).

After reaming of the acetabular cavity, insertion of the acetabular component was guided by a laser mounted on the introducer. Both the introducer and the pelvic reference laser projections were aligned to converge on a single point on the wall, ensuring the intended inclination and anteversion. Final positioning was confirmed by comparing the visibility of native bone above and below the component rim with the etched reference markings on the 3D-printed model (Fig. 4d).

Once the acetabular implant was secured in the planned orientation, femoral preparation commenced with the lower limb placed in hyperextension and external rotation without traction. After the femoral canal was prepared, the femoral stem was inserted.

For the conventional group, only X-ray examinations were conducted, and digital 2D preoperative planning was performed via the MediCAD software system (mediCAD; Hectec GmbH, Altdorf, Germany). Templates with a consistent magnification factor of 1.15 were used for the planning process, and the selected components were manually drawn onto the films. Each surgery was performed via the DAA. The prosthetic components were impacted using the freehand technique, relying solely on conventional methods and anatomical landmarks such as the transverse acetabular ligament or the anterior and posterior walls of the acetabulum.

### Outcomes

The primary outcomes included operating time (skin incision to closure) and intraoperative blood loss (based on suction canister volume after deduction of irrigation fluid) and were compared between the PSI and conventional groups.

### Data analysis

Statistical analysis was performed with XLSTAT 2022.4 (Addinsoft, France). Continuous variables are reported as the means with ranges. Normality and heteroskedasticity were assessed via Shapiro‒Wilk and Levene’s tests, respectively. The Mann‒Whitney U test was used to compare blood loss and operative time between the groups. *P* < 0.05 was considered to indicate statistical significance.

## Results

Two hundred patients were included in the study and were evenly divided into two groups: one who underwent PSI with 3D CT-scan-based preoperative planning and the other who underwent conventional surgery with 2D X-ray-based preoperative planning. The two groups did not significantly differ in demographic or clinical characteristics, including age, sex, body mass index, or implant size (Table 1).

**Table 1:** Baseline characteristics of patients

	PSI*	Conventional	*P* value
	*N* = 100	*N* = 100	
**Baseline characteristics**			
**Age** (years), mean (range)	63 (45–85)	66 (46–85)	0.25
**Male sex**, No. (%)	39 (39%)	50 (50%)	0.15
**Side**,** right**, No (%)	51 (51%)	50 (50%)	0.15
**Height (cm)**, mean (range)	169 (151–186)	168 (150–180)	0.595
**Weight (kg)**, mean (range)	74.6 (43–107)	74.0 (46–120)	0.72
**BMI (kg/m2)**, mean (range)	22.1 (14.2–32.3)	21.8 (17.4–37.4)	0.56
**Stem size**, size (%)	1 (3), 2 (6), 3 (23), 4 (27), 5 (18), 6 (14), 7 (7), 8 (2)	1 (1), 2 (8), 3 (25), 4 (16), 5 (22), 6 (11), 7 (12), 8 (5)	0,4
**Cup size**, size (%)	44 (1), 46 (12), 48 (15), 50 (24), 52 (29), 54 (9), 56 (7), 58 (3)	44 (3), 46 (15), 48 (20), 50 (19), 52 (25), 54 (15), 56 (2), 58 (1)	0,22

* PSI = patient-specific instrumentation

**Fig. 5:**
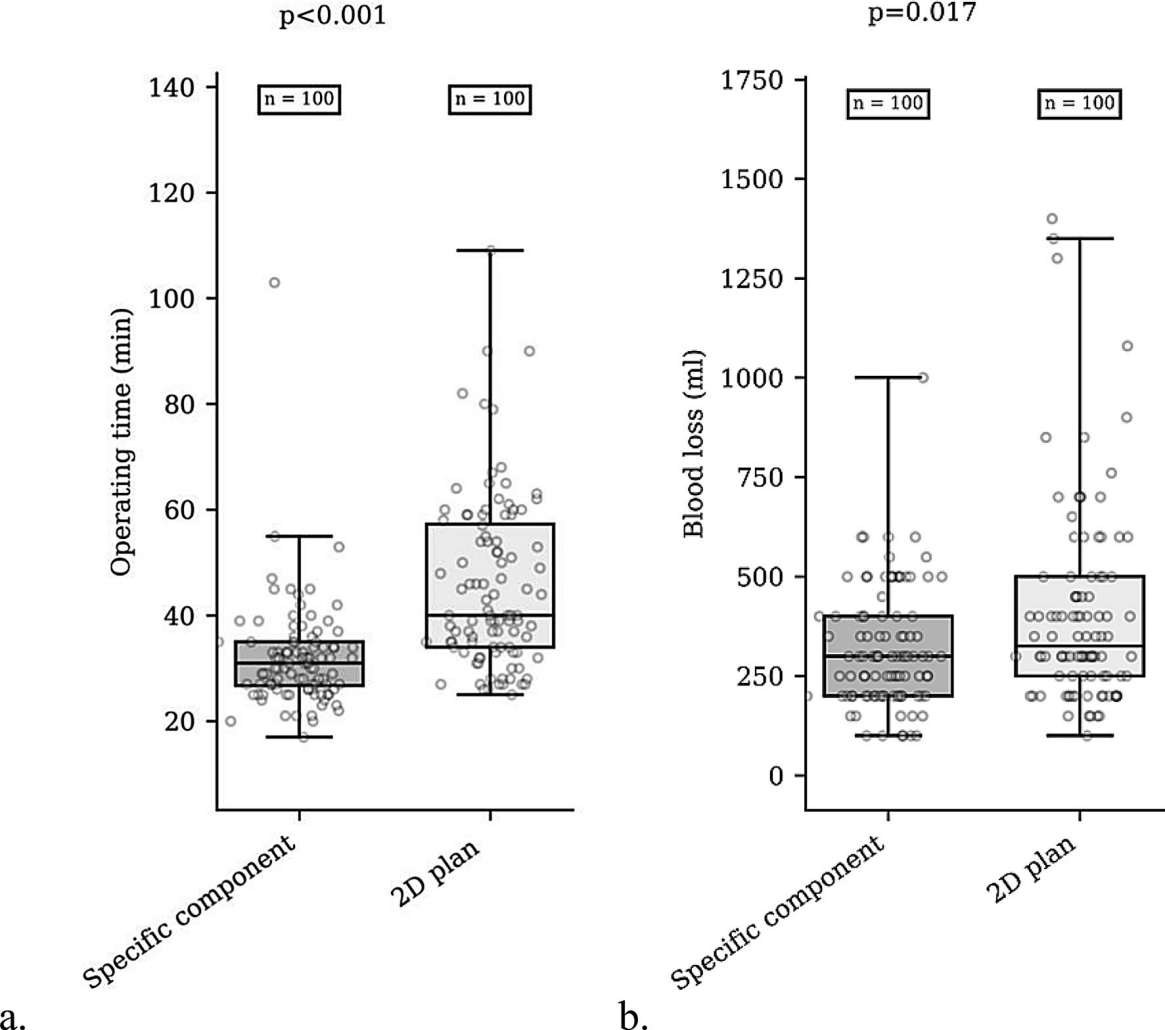
Box plot of the distribution of operating time and blood loss with PSI and 2D plan. (**a**) Comparison of operating times between the PSI and 2D planning groups (**b**) Comparison of blood loss between the PSI and 2D planning groups

Compared with the conventional group, the PSI group demonstrated significantly shorter operating times (31.9 min vs. 47.5 min, *p* < 0.001) and less blood loss (319 mL vs. 407 mL, *p* = 0.017) (Table 2). The median operating time (min) was 31.0 (IQR 8.25) in the group who underwent PSI and 3D planning and 40.0 (IQR 23.25) in the group who underwent conventional surgery with 2D planning (median Δ=-9.0; *p* < 0.001) (Fig. 5a).

**Table 2:** Comparison of operating time and blood loss between the two groups

	PSI*	Conventional	*p* value
	*n* = 100	*n* = 100	
**Operating time**			
**Minutes**, mean (range)	31,9 (18–103)	47,5 (25–109)	**< 0**,**001**
**Blood loss**			
**Millilitres**, mean **(**range)	319 (100–1000)	407 (100–1400)	**0**,**017**

* PSI = patient-specific instrumentation

The median blood loss (ml) was 300.0 (IQR 200.0) in patients in the PSI group and 325.0 (IQR 250.0) in those in the conventional surgery group (median Δ = 25.0; *p* = 0.017) (Fig. 5b).

## Discussion

The results of the present study, one of the few comparing operation time and blood loss between PSI and conventional techniques, demonstrate the effectiveness of PSI for cementless THA via the DAA. Compared with the conventional method, PSI was associated with a shorter surgery duration and reduced blood loss.

Holzer et al. [31] evaluated the accuracy of 3D CT-based preoperative planning for cementless THA and reported exact predictions of implant size for 42% of the femoral components and 37% of the acetabular components. When considering predictions within one size, the accuracy improved significantly to 87% for the femoral components and 78% for the acetabular components [31]. Sariali et al. [8] compared 2D and 3D preoperative planning for 30 patients each who underwent THA via the anterior approach by a single surgeon with different stems. The prediction accuracies for the stem and cup sizes in the 3D group were 100% and 96%, respectively [8]. Moreover, the accuracy of preoperative planning may be influenced by the size of the femoral stem. Jung et al. [32] reported higher accuracy rates for femoral stems with a rectangular design in their study. A systematic review and meta-analysis comparing 2D and 3D preoperative planning in cementless HA demonstrated that 3D planning offers superior precision in predicting both cup and stem sizes. Specifically, 3D planning achieved an accuracy of 96.92% for the cup size and 94.72% for the stem size versus 87.14% and 86.28%, respectively, for 2D planning. Additionally, 3D planning provided a detailed three-dimensional view of the patient’s anatomy, enhancing the accuracy in assessing the stem offset [33]. The improved precision achieved with 3D planning and guiding systems minimizes the need for identifying multiple intraoperative landmarks, allowing more confident placement of the stem and cup during surgery. This in turn reduces the likelihood of compromise, ultimately decreasing the duration of surgery. Similarly, the goal of PSI is to improve cup placement accuracy. In a randomized clinical trial, Small et al. [34] evaluated a different PSI technique for THA performed through a direct lateral or posterior approach. They reported significant improvements in both precision and accuracy when the desired component alignment was achieved; however, these benefits were accompanied by increased blood loss and surgery time. The PSI approach demonstrated a deviation of − 0.2° (SD 6.9°) compared with − 6.9° (SD 8.9°) in the standard (STD) group (*P* = 0.018); moreover, the PSI method achieved a mean alignment of 18.5° (SD 7.8°), which was significantly closer to the target than the alignment achieved with the STD group 28.4° (SD 7.9°) (*P* < 0.001) [32]. In a randomized controlled trial involving 64 patients equally distributed between the OPS and standard groups, Thomas et al. [35] reported that acetabular anteversion within 10° of the planned value was achieved in 96% and 76% of the patients, respectively.

Among studies analysing the operative duration associated with new planning technologies, Small et al. [34] reported a longer operative time in the PSI group than in the conventional group [95.0 min (range, 76.0–114.0) versus 88.0 min (range, 72.0–110.0), respectively]. They also reported greater blood loss in the PSI group, with 200 ml (range, 150–250) compared with 150 ml (range, 150–200) in the conventional group; however, neither of these differences was statistically significant [34]. Jin et al. [36] conducted a study evaluating the impact of PSI-assisted surgery for femoral stem implantation on operative time and intraoperative blood loss, and their results revealed no significant change in either parameter with respect to conventional techniques. These conclusions were further supported by the meta-analytical findings of Constantinescu et al. [37], who identified no significant differences in either operative duration or blood loss across studies comparing PSI-assisted surgery with conventional techniques. In their analysis, which included data from nine studies comprising a total of 533 THAs (274 controls and 259 PSI-assisted surgeries), no significant difference in operative duration was observed between the groups, with a mean difference of 2.03 min (95% CI, − 4.63 to 8.68 min; *p* = 0.55), suggesting that the addition of PSI did not substantially affect the time required for surgery. Similarly, Constantinescu et al. [37] also reported no significant difference in intraoperative blood loss between the two groups in their meta-analysis, with a mean difference of − 8.25 mL (95% CI, − 41.27 to 24.78 mL; *p* = 0.62) across seven studies including 413 THAs (214 controls and 199 PSI-assisted surgeries). Thomas et al. [35] reported an increased surgical time in the OPS group, with a mean difference of 8 min with respect to the standard group. However, the surgeries were performed via a posterior approach [35], and the operative time for this approach is reportedly shorter than that for the anterior approach [38].

Our analysis demonstrated that the PSI group had a significantly shorter operating time (31.9 min vs. 47.5 min, *p* < 0.001) and less blood loss (319 mL vs. 407 mL, *p* = 0.017) than did the conventional group. Similarly, a comparison of the median values showed that patients in the PSI group had a significantly lower operating time [31.0 min (IQR 8.25)] than the conventional surgery group [40.0 min (IQR 23.25)] (median Δ = −9.0, *p* < 0.001). Blood loss in the PSI group was also lower, with a median of 300.0 mL (IQR 200.0) versus 325.0 mL (IQR 250.0) in the conventional group (median Δ = 25.0, *p* = 0.017). Placement of the patient in the supine position on an orthopaedic table leads to significant variability in pelvic positioning, making it more challenging to manually place the acetabular implant and achieve the desired orientation [39]. This requires the identification of multiple landmarks, such as the transverse acetabular ligament, the alignment of the anterior superior iliac spines beneath the surgical drape, or the anatomy of the acetabular cavity. These extensive pelvic positioning verification methods are time-consuming, whereas the use of a laser guide eliminates this constraint, potentially explaining, in part, the reduction in operative time.

The results of the present study suggest that in contrast to the lack of significant changes reported previously, the use of PSI in our cohort not only shortened the operative time but also reduced blood loss. This may reflect the improved surgical precision provided by PSI, which could enable a more efficient and controlled procedure, ultimately reducing both the operating time and the amount of intraoperative bleeding. Additionally, the use of the DAA for THA may have contributed to the reductions in various durations, as previous studies have reported significantly shorter surgery durations for experienced surgeons between DAA-THA and posterior-approach THA [40]. These findings underscore the potential benefits of PSI surgery in improving both surgical efficiency and minimizing blood loss, making it a promising alternative to conventional methods. In addition to reducing infection risk, shorter operating times are also important in lowering operating room (OR) costs [41]. According to a study by Childers et al. [39], which estimated OR costs at $36 to $37 per minute, this reduction would translate to savings of approximately $360–$370 per patient with the use of PSI via the DAA.

The present study has several limitations that warrant consideration. First, while this was a retrospective study, the data analysed were collected prospectively.

Second, the study focused exclusively on stems with straight, tapered designs featuring a quadrangular cross-section and full coatings. While this uniformity ensured consistency within the dataset, it also limits the generalizability of the findings to other stem geometries, textures, and coatings. Variations in these design characteristics may influence planning accuracy, surgical outcomes, and implant performance. Therefore, further research incorporating stems with diverse geometries, surface textures, and coating types would be of significant interest.

Moreover, while the use of 3D planning and dedicated guides can help shorten the surgical procedure, operative time also depends on patient-specific characteristics, such as body weight, hip stiffness, and the size of the implants, which may require the use of additional reamers or femoral broaches. These factors can explain outliers with operative times reaching up to 100 min. However, there were no differences between the two cohorts in terms of sex, BMI, or implant size that could have influenced operative duration.

Another limitation is that blood loss assessment was limited to intraoperative volume estimation and did not include total blood volume loss calculation (e.g., using the Mercuriali formula). Postoperative hemoglobin levels were not routinely measured in the absence of significant intraoperative bleeding, in accordance with our institutional practice. This may have underestimated the overall blood loss.

Finally, although 3D templating is performed using low-dose CT scans [42], it remains associated with higher radiation exposure and increased costs. Therefore, studies assessing its cost-benefit ratio are warranted.

## Conclusion

The use of PSI and technological assistance demonstrated advantages in reducing both operating time and blood loss, with an average reduction of over 10 min. Furthermore, an analysis of the standard deviations indicates improved consistency in operating times with the use of custom guides than with conventional instrumentation. These findings can be attributed to the enhanced accuracy of preoperative planning and intraoperative procedures facilitated by 3D planning and custom guides, minimizing the need for intraoperative adjustments, streamlining the surgical workflow, and providing greater confidence in accurately positioning the stem and cup. These benefits highlight the potential of PSI and advanced surgical technologies in improving both the efficiency and reproducibility of THA surgery via the DAA.

## Data Availability

The datasets used and/or analysed during the current study are available from the corresponding author upon reasonable request.

## Abbreviations

THA: Total hip arthroplasty
PSI-THA: Patient-specific instrumentation for total hip arthroplasty
DAA: Direct anterior approach
PSI: Patient-specific instrumentation
LSZ: Lewinnek safe zone
